# Antibody-Dependent Cellular Cytotoxicity Elicited by the Antibodies Against the E120R Protein of African Swine Fever Virus

**DOI:** 10.3390/vaccines13090934

**Published:** 2025-09-01

**Authors:** Shengmei Chen, Jing Lan, Zhanhao Lu, Jia Li, Caoyuan Ma, Rui Luo, Qiang Fu, Yuan Sun, Tao Wang, Hua-Ji Qiu

**Affiliations:** 1 State Key Laboratory for Animal Disease Control and Prevention, National African Swine Fever Para-Reference Laboratory, National High Containment Facilities for Animal Diseases Control and Prevention, Harbin Veterinary Research Institute, Chinese Academy of Agricultural Sciences, Harbin 150069, China; 2 College of Life Science and Engineering, Foshan University, Foshan 528231, China

**Keywords:** African swine fever, African swine fever virus, antibodies, E120R protein, antibody-dependent cellular cytotoxicity

## Abstract

**Background/Objectives:** African swine fever (ASF) is a disease of domestic pigs and wild boar caused by African swine fever virus (ASFV), in which infection often leads to high morbidity and mortality. Although subunit and mRNA vaccines based on protective antigens have been explored for ASFV, their protective efficacy remains insufficient for practical ASF control, highlighting the need to identify new potential antigens capable of inducing more potent and broadly protective immune responses. Previously, we found that the antibodies against the ASFV E120R protein (pE120R) could significantly inhibit virus replication in primary porcine alveolar macrophages (PAMs). However, it is not yet known whether anti-pE120R antibodies can induce antibody-dependent cellular cytotoxicity (ADCC). **Methods:** In this study, we analyzed the conservation and immunogenic features of pE120R and established an HEK293T cell line with stable expression of pE120R as target cells (HEK293T-pE120R). Additionally, a co-culture system comprising target cells and peripheral blood mononuclear cells (PBMCs) was established to evaluate the ability of the anti-pE120R antibodies to induce ADCC as measured by lactate dehydrogenase (LDH) release assays. **Results:** The results showed that pE120R is highly conserved among different ASFV genotypes and contains multiple B-cell and T-cell epitopes. Importantly, LDH release assays demonstrated that anti-pE120R antibodies triggered NK cell-mediated ADCC. Notably, ASFV replication in HEK293T-pE120R cells was not promoted. **Conclusions:** In summary, pE120R was associated with antibody production in a cytotoxicity assay. The ability of this antigen to induce protective immunity, if any, requires further evaluation *in vivo*.

## 1. Introduction

African swine fever (ASF) is a highly contagious viral disease caused by African swine fever virus (ASFV), affecting both domestic pigs and wild boar with high mortality rates [1]. Due to the genetic diversity among ASFV genotypes and concerns regarding reversion to virulence or recombination, live-attenuated vaccines (LAVs) face challenges in providing broad cross-protection, limiting their large-scale commercial application. Despite the approval of two ASF LAVs in Vietnam, questions remain regarding their safety and protective efficacy [2,3]. Novel vaccine platforms based on protective antigens, such as subunit vaccines, mRNA vaccines, and viral vector vaccines, have shown limited efficacy, largely due to the incomplete identification of protective antigens [4]. Therefore, there is an urgent need to identify antigens capable of inducing robust protective immune responses, which could serve as promising targets for the development of next-generation ASF vaccines.

The SARS-CoV-2 pandemic has accelerated the development of mRNA vaccines [5]. These advances have inspired research into applying similar approaches to other pathogens, including ASFV, though no ASFV mRNA vaccines are yet commercially available. Protective immunity mediated by antigens can be achieved through three major mechanisms. First, although no potent neutralizing antibodies against ASFV have been identified, certain non-neutralizing antibodies, such as anti-CD2v antibodies, can inhibit key viral processes like hemadsorption and provide protection. Second, antigen-specific CD8^+^ T-cells can be activated to eliminate virus-infected cells. Third, antigens can stimulate synergistic interactions between humoral and cellular immunity. This synergy is exemplified by mechanisms such as antibody-dependent cellular cytotoxicity (ADCC), complement-dependent cytotoxicity (CDC), and antibody-dependent cellular phagocytosis (ADCP) [6,7]. Among these, ADCC serves as a key model for understanding how antibodies bridge innate and adaptive immune responses. It involves the specific binding of the Fab region to antigens on the target cell surface, followed by the recognition of the Fc region by Fc*γ*RIIIa (CD16a) on NK cells, leading to NK cell activation and the lysis of the infected cell.

The E120R protein (pE120R) is a capsid protein encoded by ASFV, classified as a structural protein. It is highly conserved among ASFV strains and plays a key role in viral transport through interactions with host microtubules. Previous studies have shown that pE120R is present on the surface of viral particles, suggesting its potential exposure on infected cells [8]. Notably, it has been shown that the pE120R of ASFV inhibits the cGAS/STING signaling pathway and interferes with interferon production, thereby weakening the antiviral response in infected cells and facilitating viral replication [9]. In addition, our team recently observed that anti-pE120R antibodies partially inhibit ASFV replication *in vitro* in primary porcine alveolar macrophages (PAMs), suggesting that pE120R may contribute to protective immunity and warrants further investigation as a potential antigen. Building on this finding, the present study aimed to generate target cells stably expressing pE120R on their surface and evaluate whether anti-pE120R antibodies can induce ADCC.

## 2. Materials and Methods

### 2.1. Anti-pE120R Antibodies and Virus Strains

The recombinant pE120R with a His-tag at the N-terminus was expressed in *Escherichia coli* BL21 (DE3) using the pCold-I vector. Following induction, the cells were lysed by sonication and the soluble fraction was incubated with Ni^+^ Sepharose High Performance (Cytiva, Marlborough, MA, USA, 17526802) to capture the His-tagged protein. The bound protein was eluted with 250 mM imidazole in Tris-HCl buffer.

To generate anti-pE120R antibodies, purified recombinant pE120R was used as the immunogen. Four-week-old, healthy, ASFV-negative piglets were intramuscularly immunized with 100 µg of pE120R in 1 mL of phosphate-buffered saline (PBS) containing 15% MONTANIDE ISA 15A VG. Booster doses were administered at 14 and 28 days after the initial immunization. Serum samples were collected at day 42. The ASFV strain adapted to HEK293T cells, designated as ASFV-P121, was obtained through previously established protocols [10].

### 2.2. pE120R Sequence Conservation Analysis Across ASFV Genotypes

Sequence alignment analysis was performed to assess the sequence variability of the ASFV pE120R and to identify conserved regions across different genotypes. Nine representative ASFV strains were retrieved from the NCBI database, including four genotype II, three genotype I, and two recombinant (genotype I/II) strains. Collected from diverse geographic regions, these strains span multiple sampling times and provide a broad representation of circulating ASFV variants (Table 1). All sequences were manually curated and subjected to multiple sequence alignment using the MegAlign module in the DNAStar Lasergene software (version 7.1).

### 2.3. Bioinformatics Analysis of the Antigenic Features of the pE120R

To evaluate the immunogenic potential of pE120R, we conducted a systematic bioinformatics analysis of its antigenicity, hydrophilicity, secondary structure, and potential B-cell and T-cell epitopes. The pE120R reference amino acid sequence was obtained from the ASFV HLJ/18 strain (GenBank accession no. MK333180.1). Antigenic indices were calculated using the Jameson–Wolf algorithm in the Protean module of the DNAstar Lasergene software (version 7.1)) and secondary structure elements (*α-*helices, *β-*sheets, turns, and random coils) were predicted. Hydrophobicity analysis was performed using the Kyte–Doolittle hydropathy scale via the ExPASy ProtScale tool (https://web.expasy.org/protscale/, accessed on 20 June 2025). A window size of 9 amino acids was used with uniform residue weighting across the window, following the linear weight model at a 100% edge weight. Predictions for SLA-A*01:01 were performed using NetMHCpan 4.1 to identify potential CD8^+^ T-cell epitopes based on 9-mer peptides. Due to limited experimental data for SLA alleles, the prediction was based on the closest available allele, SLA-104:01, with an allele distance of 0.229. The analysis was conducted using the IEDB resource at http://tools.iedb.org/mhci/. CD4^+^ T-cell epitopes were predicted using NetMHCIIpan 4.1 for HLA-DRB1*01:01, with 15-mer peptides showing a percentile rank < 1% considered as strong binders [11,12]. Linear B-cell epitopes were predicted using BepiPred-2.0 (IEDB) with a threshold of 0.5, based on a random forest model trained on antibody–antigen crystal structures (Table 2).

### 2.4. Design of a Lentiviral Expression Vector

Based on the sequence of the pE120R from the ASFV HLJ/18 strain, a signal peptide sequence was added to the N-terminus, and the transmembrane domain of the vesicular stomatitis virus glycoprotein G (VSV-G) along with a 6× His tag were appended to the C-terminus. The optimized gene was synthesized by GENEWIZ from Azenta Life Sciences (Suzhou, China). Subsequently, the optimized gene was cloned into the lentiviral vector pLVX-Puro, which contains the ZsGreen reporter gene. The resulting construct was designated as pLVX-E120R.

### 2.5. Lentivirus Packaging and Infection

Human embryonic kidney 293T (HEK293T) cells were cultured in Dulbecco’s modified Eagle’s medium (DMEM) (Gibco, New York, NY, USA, 612320) and supplemented with 10% fetal bovine serum (Gibco, New York, NY, USA, A5669701). The cells were maintained at 37 °C in a humidified atmosphere containing 5% CO_2_. HEK293T cells were seeded at 10^6^ cells/mL in T25 flasks and cultured for 16–24 h until reaching 70% to 80% confluence. The cells were transfected with pLVX-E120R (6 μg), psPAX2 (4 μg), and pMD2.G (2 μg) using 36 μg of linear 25-kDa polyethyleneimine (PEI) (Polysciences, Warrington, PA, USA, 23966-2) in Opti-MEM reduced serum medium (Thermo Fisher Scientific, Waltham, MA, USA, 31985070). The virus-containing supernatants were collected at 48 h post-transfection (hpt) for target cell infection. The lentiviral supernatants were mixed 1:1 with fresh medium containing 8 μg/mL polybrene (G-CLONE, Shanghai, China, CC3110) and added to T25 flasks containing HEK293T cells at 80% confluence.

### 2.6. Western Blotting

Cell lysates were prepared on ice using RIPA lysis buffer (Sigma-Aldrich, Darmstadt, Germany, R0278) containing a protease inhibitor cocktail (Roche, Basel, Switzerland, 4693116001). After clarification, the lysates were mixed with SDS-PAGE sample buffer (Solarbio, Beijing, China, P1040), denatured by boiling, and subjected to electrophoresis on SDS-PAGE gels. Proteins were then transferred onto polyvinylidene fluoride (PVDF) membranes, which were blocked for 2 h at room temperature with 5% non-fat milk in Tris-buffered saline supplemented with 0.05% Tween 20 (TBST). Following blocking, membranes were incubated with specific primary and corresponding secondary antibodies. Immunoreactive bands were detected using the Odyssey imaging system.

### 2.7. Screening and Characterization of an HEK293T Cell Line Stably Expressing pE120R

ZsGreen fluorescence was visualized using a fluorescence microscope (Thermo Fisher Scientific, Waltham, MA, USA). ZsGreen-positive cells were enriched by fluorescence-activated cell sorting (FACS) using a MoFlo XDP flow cytometer (Beckman Coulter, Indianapolis, IN, USA), followed by the selection and subsequent expansion of cell clones. Genomic DNA was extracted using a genomic DNA extraction kit (Tiangen, Beijing, China, DP304). Specific primers targeting the *E120R* gene were designed and used for PCR amplification (Table 3). In addition, Western blotting analysis was performed to evaluate the pE120R expression level using an anti-His antibody (Solarbio, Beijing, China, K200060M) and an anti-*β*-actin antibody (Abclonal, Wuhan, China, A12289) as the internal control.

### 2.8. Laser Confocal Scanning Microscopy

HEK293T cells were seeded onto 20 mm glass-bottom culture dishes and allowed to adhere overnight to ensure optimal spreading and monolayer formation prior to immunostaining. At designated time points post-treatment or transfection, the cells were fixed with 4% paraformaldehyde (PFA) in PBS for 20 min at room temperature to preserve cellular architecture. Following fixation, the cells were washed with PBS and permeabilized with 0.1% triton X-100 for 30 min at 25 °C to enable antibody access to intracellular antigens. To minimize non-specific binding, samples were blocked with 5% bovine serum albumin (BSA) in PBS for 30 min under ambient conditions [13]. After blocking, the cells were incubated with a mouse anti-His primary antibody at room temperature for 1 h, followed by extensive washing and subsequent incubation with an Alexa Fluor 647-conjugated goat anti-mouse IgG secondary antibody (Thermo Fisher Scientific, Waltham, MA, USA, A-21235) for 1 h in the dark to ensure fluorescence stability. Nuclear DNA was counterstained with DAPI (Solarbio, Beijing, China, ID2250) for 10 min to facilitate cellular localization and a morphological assessment. The excess stain was removed through multiple PBS washes and samples were immediately imaged using a confocal laser scanning microscope (Carl Zeiss, Oberkochen, Germany) equipped with a 63× oil immersion objective (numerical aperture 1.4). Optical sections were acquired using appropriate laser wavelengths and collected as *z*-stacks, which were then processed into maximum-intensity projections in the *x*–*y* plane to assess the three-dimensional distribution and membrane localization of pE120R.

### 2.9. Membrane Fractionation

Membrane proteins were extracted from HEK293T-pE120R cells using a membrane and cytoplasmic protein extraction kit (Beyotime, Shanghai, China, P0033), according to the manufacturer’s instructions. HEK293T cells expressing pE120R were harvested, resuspended in cytoplasmic lysis buffer, incubated on ice for 10 min, and lysed by repeated freeze–thaw cycles. The lysates were centrifuged at 14,000× *g* for 30 min at 4 °C, the resulting supernatants were designated as the cytoplasmic protein fraction. The pellet was resuspended in 200 µL of membrane protein extraction reagent, vortexed briefly, and incubated on ice. This resuspension step was repeated once. Following another centrifugation, the supernatants were collected as the membrane protein fraction and subjected to Western blotting analysis to check the pE120R expression.

### 2.10. ADCC Measurement

To evaluate the ADCC activities mediated by anti-pE120R, HEK293T cells stably expressing pE120R were used as the target cells. A total of 50 µL of the target cell suspension (10^6^ cells/mL) was seeded into a 96-well culture plate and incubated with anti-pE120R antibodies at 37 °C for 2 h. Subsequently, 50 µL of freshly isolated porcine peripheral blood mononuclear cells (PBMCs), serving as effector cells, were added at a concentration of 10^7^ cells/mL, establishing an effector-to-target (E:T) ratio of 10:1. PBMCs were obtained from EDTA-anticoagulated whole blood via density gradient centrifugation using a porcine-specific lymphocyte isolation kit (Solarbio, Beijing, China, P6230) and used directly without cryopreservation or thawing to preserve the cellular viability and function. The co-cultures were then incubated under standard conditions (37 °C, 5% CO_2_) for 5 h. Following incubation, supernatants were carefully collected and centrifuged to remove cellular debris. LDH release, as a marker of membrane integrity loss and cell death, was quantified using the CytoTox 96 non-radioactive cytotoxicity assay kit (Promega, Madison, WI, USA, G1780) in accordance with the manufacturer’s protocols. The percentage of specific cytotoxic activity was calculated using the following formula:

$$Cytotoxicity=\frac{\left(Experimental-Effector\;Spontaneous-Target\;Spontaneous\right)}{\left(Target\;Maximum-Target\;Spontaneous\right)}\times 100\%$$

### 2.11. Effects of pE120R on ASFV Replication

To investigate the impact of pE120R expression on ASFV replication, HEK293T cells and HEK293T-pE120R cell lines were infected with the ASFV-P121 strain at a multiplicity of infection (MOI) of 1 [14]. Following infection, the cells were incubated under standard culture conditions (37 °C, 5% CO_2_) and harvested at 24 and 48 h post-infection (hpi). Viral protein expression was assessed by Western blotting using a mouse monoclonal antibody against the conserved ASFV p72 protein, used as a marker of viral replication [15]. Rabbit anti-GAPDH (ABclonal, Wuhan, China, A12289) served as a loading control. Fluorescent detection was carried out using IRDye 800CW-conjugated goat anti-mouse IgG (LI-COR Biosciences, Lincoln, NE, USA, 926-32212) and goat anti-rabbit IgG (LI-COR Biosciences, Lincoln, NE, USA, 926-32211) secondary antibodies, and signals were visualized using the Odyssey infrared imaging system (LI-COR Biosciences). In parallel, total genomic DNA was isolated from infected cells using the Tiangen nucleic acid extraction and purification kit (Tiangen, Beijing, China, DP315), and the ASFV genome copies were quantified by real-time quantitative PCR (qPCR).

### 2.12. Statistical Analysis

Statistical analysis was performed using an unpaired Student’s *t* test to compare group means (GraphPad Prism 9.5.0, San Diego, CA, USA). Data are presented as the mean ± SD of at least three independent experiments. *p* ≤ 0.05 was considered statistically significant (* *p* < 0.05, ** *p* < 0.01, *** *p* < 0.001).

## 3. Results

### 3.1. pE120R Is Conserved Among Various ASFV Genotypes

**Figure 1:**
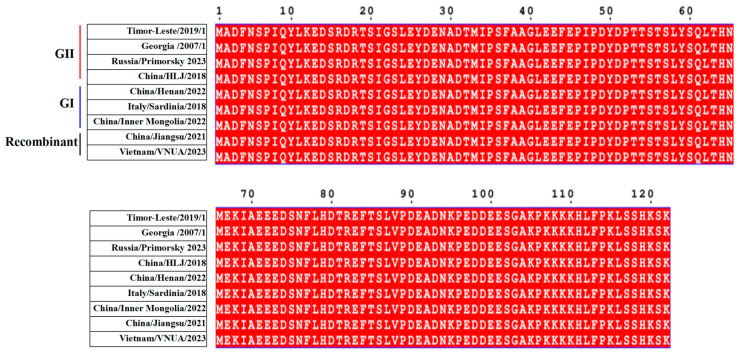
Homology analysis of pE120R. Amino acid sequences of pE120R from representative ASFV isolates were aligned to identify conserved and variable regions. The alignment includes genotypes I and II strains and circulating genotype I/II recombinant strains. Identical residues across sequences are highlighted in red.

The amino acid (aa) sequences of pE120R from genotypes I, II and I/II recombinant strains of ASFV were retrieved from GenBank. Nine representative isolates that have been circulating in Eurasia in recent years were selected for alignment analysis (Figure 1, Table 1). The results showed that pE120R is highly conserved across different ASFV genotypes.

### 3.2. Immunogenicity and Epitope Prediction Analysis

**Figure 2:**
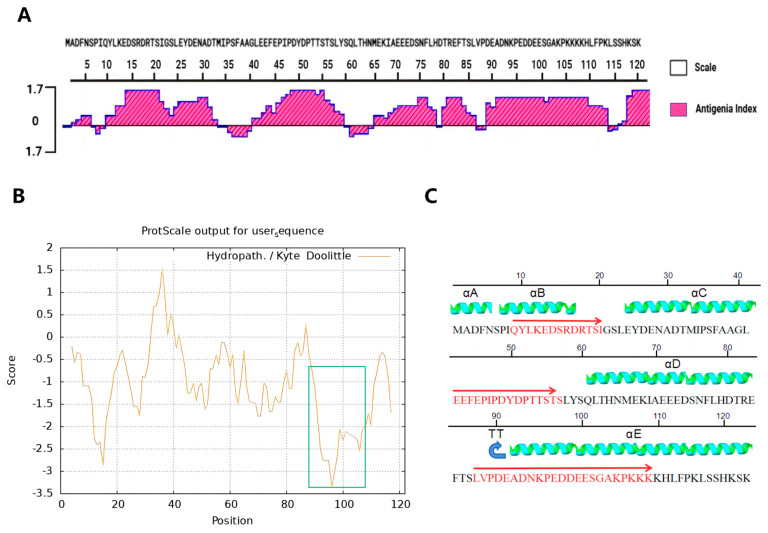
Immunogenicity and epitope prediction analysis. (**A**) Antigenic index of the pE120R protein. The antigenic index is plotted against the amino acid position. Peaks indicate regions with higher antigenicity. (**B**) Hydrophobicity analysis of pE120R. The hydrophobic regions were calculated using the Kyte–Doolittle method. The green-highlighted region (aa 90–110 region) corresponds to a hydrophilic segment with negative hydrophobicity scores. (**C**) The 2D structure of pE120R. B-cell epitope regions highlighted in red. Alpha helices are shown in green and turn structures are indicated by “TT” in blue arrows. The positions of B-cell epitopes are specifically denoted by red arrows.

To evaluate the potential of pE120R as a protective antigen, we systematically analyzed its immunogenicity and reactogenicity. DNAstar software (version 7.1) predicted that pE120R contains multiple high-antigenicity regions within the aa 90–110 region, including two charge-enriched zones. One contains predominantly negatively charged D and E residues, and the other mainly positively charged K, R, and H residues. These highly concentrated charges are conducive to electrostatic interactions, indicating strong immunogenicity (Figure 2A). Furthermore, epitope prediction using the (IEDB) database revealed multiple overlapping immunodominant epitopes within the aa 90–110 region of pE120R, including CD4^+^ T-cell epitopes (^80^DTREFTSLVPDEADN^94^, ^81^TREFTSLVPDEADNK^95^, ^82^REFTSLVPDEADNKP^96^, and ^79^HDTREFTSLVPDEAD^93^) and a B-cell epitope (^87^LVPDEADNKPEDDEESGAKPKKK^109^), suggesting strong immunogenicity in this region. In addition, multiple CD8^+^ T-cell epitopes and B-cell epitopes were predicted across different regions of the protein, further supporting the potential of pE120R as a protective antigen (Table 2). Meanwhile, the aa 90–110 region is predicted to be highly hydrophilic and likely exposed on the protein surface, which may facilitate the binding of antibodies to the antigen (Figure 2B). A secondary structure prediction of pE120R using the DNAstar software (version 7.1) revealed that the antigen is rich in *α*-helical structures. Further analysis indicated that some immunodominant epitopes significantly overlap with these helical regions, suggesting that these domains not only exhibit high conformational stability, but also represent potential immunogenic targets (Figure 2C).

### 3.3. Generation of a Stable HEK293T Cell Line Expressing pE120R

**Figure 3:**
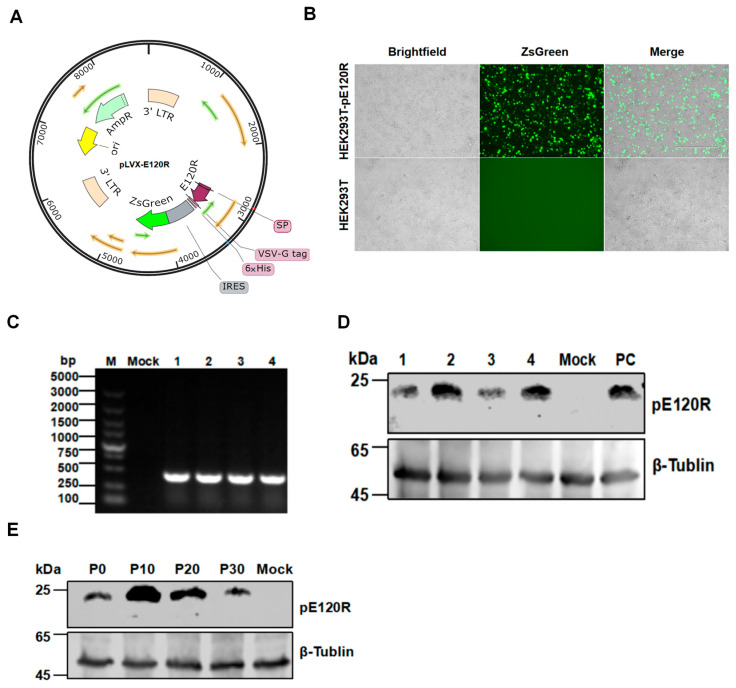
Establishment and characterization of the stable cell line HEK293T-pE120R. (**A**) Schematic diagram of the lentiviral vector harboring *E120R*. The vector includes elements for expression, detection, and purification, with ZsGreen fluorescence and a C-terminal 6× His tag. (**B**) Green fluorescence in the transfected HEK293T cells. Scale bar, 20 μm. (**C**) Detection of the *E120R* gene integration in the stable cells by PCR. No signal was detected in non-transfected cells (Mock), used as the negative control. Lanes 1–4 represent four independent cell clones. (**D**) Expression of pE120R in stable cells by Western blotting analysis. HEK293T cells transfected with the pE120R expression plasmid served as a positive control (PC). (**E**) Stable expression of pE120R in the cells after serial passage by Western blotting analysis.

**Figure 4:**
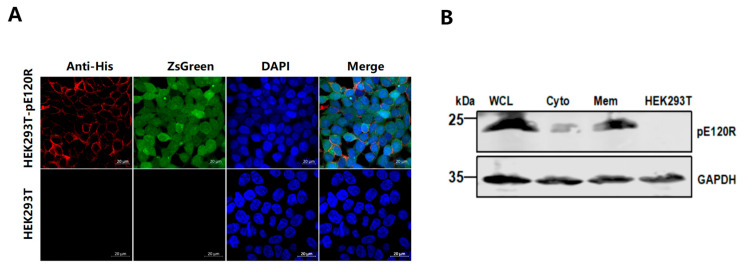
Expression of pE120R on the cell membrane. (**A**) Confocal microscopy analysis of pE120R localization on the cell membrane. Scale bar, 20 μm. Anti-His antibody was used as the primary antibody, followed by Alexa Fluor 647-conjugated goat anti-mouse IgG secondary antibody to detect pE120R localized on the cell membrane (red signal). ZsGreen, expressed stably in the cells, served as an intrinsic green fluorescent marker (green signal) without additional staining. Nuclei were stained with DAPI (blue). (**B**) The membrane localization of pE120R. Whole cell lysates (WCL), cytosolic fractions (Cyto), and membrane fractions (Mem) were prepared from HEK293T-pE120R cells and subjected to Western blotting analysis using an anti-pE120R antibody.

HEK293T cells were transduced with a recombinant lentiviral vector encoding the *E120R* gene and the ZsGreen fluorescent reporter gene (Figure 3A). Strong green fluorescence was observed at 48 h post-transduction under a fluorescence microscope (Figure 3B), indicating a high transfection efficiency. ZsGreen-positive cells were then enriched by FACS and expanded in culture for further analysis. PCR amplification confirmed the stable genomic integration of the *E120R* gene in the cell line, yielding a specific DNA fragment of approximately 400 bp (Figure 3C). Western blotting analysis with an anti-His tag antibody detected a specific protein band of approximately 25 kDa in HEK293T-pE120R cell lysates, close to the predicted molecular weight of the recombinant pE120R (Figure 3D). To evaluate the expression stability, no significant decrease in the pE120R level was observed across multiple passages from P0 to P30 (Figure 3E). Confocal microscopy showed the clear membrane localization of pE120R, while ZsGreen fluorescence confirmed the cell viability and successful viral transduction (Figure 4A). Western blotting analysis of the membrane protein fraction also detected a band of approximately 25 kDa (Figure 4B). Together, these results indicate that pE120R is correctly translated and stably expressed on the cell membrane.

### 3.4. Anti-pE120R Antibodies Mediate ADCC Activities

**Figure 5:**
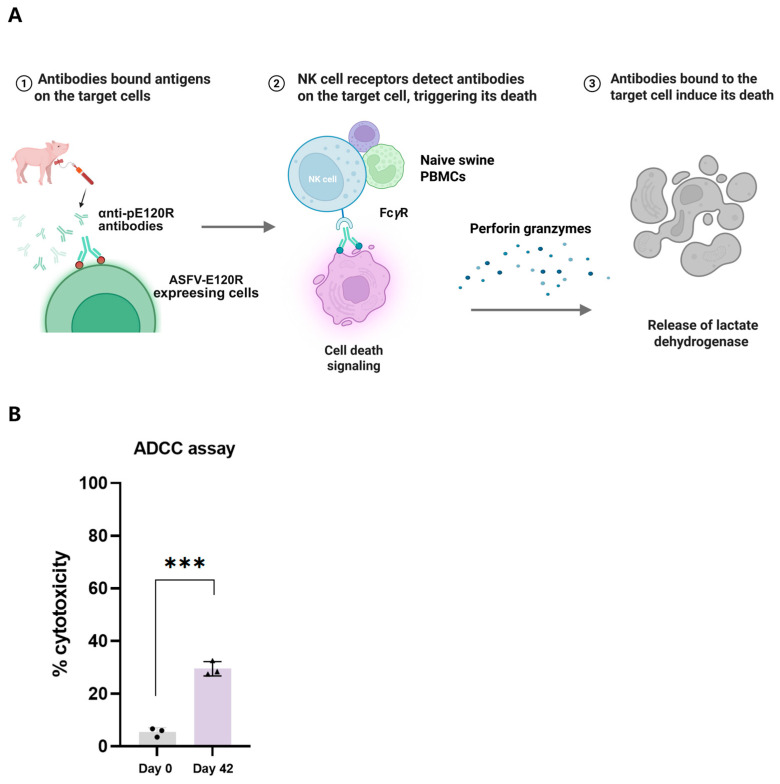
ADCC elicited by anti-pE120R antibodies. (**A**) Schematic illustration of the ADCC mechanism. Antibodies bind to pE120R expressed on the surface of target cells. The Fc region of the antibody binds to Fc*γ* receptors (Fc*γ*R) on cytotoxic effector cells, triggering the release of perforin and other cytotoxic molecules, which ultimately leads to target cell lysis. (**B**) ADCC activities induced by the sera from the pE120R-immunized pigs. Analysis of ADCC activities by measuring LDH release in the supernatant of cells overexpressing ASFV pE120R following incubation with serum collected at 0 dpi and 42 dpi, and subsequent addition of naïve swine PBMCs. Black circles represent individual data points from pigs immunized with pE120R at 0 days post immunization (dpi), and black triangles represent individual data points at 42 dpi. *** *p* < 0.001.

The functional activity of anti-pE120R antibodies was evaluated using the ADCC assay (Figure 5A). In this assay, HEK293T cells stably expressing ASFV pE120R (target cells) were incubated with anti-pE120R antibodies collected at 0 days (preimmunization) and 42 days post-immunization. Subsequently, naïve swine PBMCs were added as effector cells to form antibody–target–effector cell complexes. Cytotoxicity was assessed by measuring the LDH release in the supernatant of each well. Compared with the control group treated with preimmunization serum, the serum collected at day 42 showed a significant increase in cytotoxicity, reaching up to 29% (Figure 5B). These findings demonstrate that anti-pE120R antibodies can induce ADCC during the interaction between effector and target cells.

### 3.5. ASFV Replication Is Not Significantly Enhanced in the HEK293T-pE120R Cell Line

**Figure 6:**
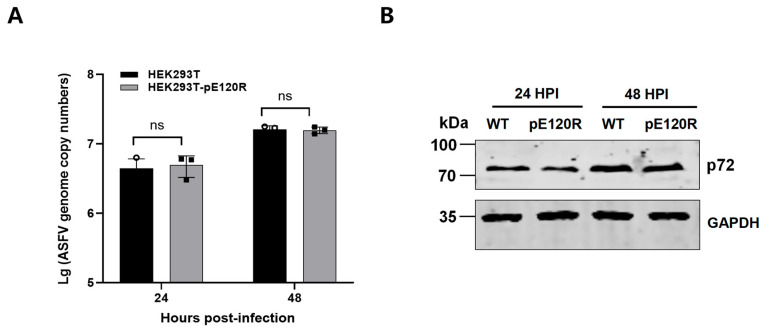
pE120R does not affect ASFV replication. (**A**) Quantification of ASFV genome copies by qPCR in HEK293T-pE120R cells at 24 and 48 hpi with ASFV. The data are presented as means ± SD from three independent experiments. Statistical analysis was performed using an unpaired *t* test; ns, not significant. (**B**) Detection of the ASFV p72 protein in HEK293T-pE120R cells by Western blotting analysis at 24 and 48 hpi. WT represents control HEK293T cells. GAPDH is the loading control.

To further evaluate the impact of the pE120R on ASFV replication, the HEK293T-pE120R cells were infected with the ASFV-P121 strain. Viral replication was analyzed by Western blotting and qPCR. qPCR analysis revealed no significant difference in viral genome copies between HEK293T-pE120R and control HEK293T cells at 24 and 48 hpi (Figure 6A). Similarly, Western blotting showed comparable levels of viral protein expression in both cell lines (Figure 6B).

## 4. Discussion

Although several ASF vaccines have shown efficacy in preventing clinical disease, none are currently available that are both safe and capable of supporting sustainable control or eradication programs. For instance, two LAVs licensed in Vietnam can reduce clinical symptoms but impair the pig reproductive performance and are ineffective against the newly emerging genotype I/II recombinant strains [2,3]. Given the limitations of LAVs, novel strategies such as subunit, mRNA, and vector vaccines are gaining attention [16,17,18]. Current vaccine research focuses on identifying antigens that can induce both antiviral replication and cytotoxic effects [19,20]. To evaluate the potential of pE120R as a vaccine target, we performed bioinformatic analysis and found pE120R to be highly conserved, thus indicating its potential as a promising target for mRNA vaccines. Immunogenicity predictions revealed that the aa 90–110 region is rich in charged residues, highly hydrophilic, contains multiple overlapping T-cell and B-cell epitopes, and partially overlaps with predicted *α*-helical structures, indicating its potential as a protective antigen.

In the evaluation of ADCC activities, three commonly used methods have been described [21], including flow cytometry-based assays [22], commercial fluorescent reporter gene systems [23,24], and the LDH release assay. The key differences among these methods lie in the strategies for target cell construction and the mechanisms used to detect cell death. The flow cytometry method assesses target cell lysis by labeling surface antigens and membrane integrity markers, such as propidium iodide (PI) or 7-AAD. However, it may miss early apoptotic events where the cell membrane remains intact [25]. Fluorescent reporter systems offer high-throughput screening with good reproducibility by measuring emitted fluorescence signals. Nevertheless, they do not directly reflect target cell lysis and typically require specific effector cells expressing Fc receptors like Fc*γ*RIIIa, along with compatible commercial kits. In contrast, the LDH release assay measures the enzymatic activity of LDH released into the culture supernatant upon cell lysis. It is straightforward, intuitive, and widely applicable [26]. Therefore, we selected this method to evaluate ADCC activities in our study. By choosing an appropriate target cell design and a reliable method for detecting cell death, we successfully assessed the ability of anti-pE120R antibodies to induce *in vitro* ADCC activities.

Our results showed that anti-pE120R antibodies induced ADCC *in vitro,* achieving a cytotoxicity level of approximately 29%. In comparison, a similar experiment targeting another outer membrane protein, p30, yielded only about 18% cytotoxicity [27]. Notably, the target cell line used in our study stably expressed the full-length pE120R rather than just processed antigenic peptides. In contrast, *in vivo* antigen-presenting cells typically present only short peptide fragments, usually consisting of around 10 amino acid residues, via MHC molecules [28]. Such a limited antigen presentation may impair antibody recognition and binding, ultimately reducing the efficiency of ADCC induction. Therefore, the ADCC activities of anti-pE120R antibodies *in vivo* may be lower than what was observed *in vitro*. This hypothesis aligns with recent findings from studies on SARS-CoV-2 [23], which suggest that antibodies showing strong ADCC activities *in vitro* may not necessarily confer equivalent immune protection *in vivo*. This highlights the importance of utilizing multiple approaches, such as CDC and ADCP, rather than relying solely on a single *in vitro* functional assay, to evaluate ASFV-specific antibody responses.

pE120R, a late-stage structural protein of ASFV, inhibits type I interferon (IFN-*α*/*β*) production [9] and plays a critical role in the transport and release of viral particles from the cytoplasm to the cell membrane [29]. Although pE120R is not essential for viral assembly or initial infection, it is crucial for efficient viral spread. To investigate whether pE120R expression alone could facilitate ASFV replication, we compared the viral replication efficiency in HEK293T cells stably expressing pE120R versus control cells. Interestingly, no significant enhancement was observed. There are several possible explanations for this result. First, pE120R may require other viral proteins or specific infection conditions such as inflammation or stress to function effectively. Second, the overexpression of pE120R alone might disrupt the viral protein balance or assembly [30]. Third, HEK293T cells are derived from human embryonic kidney tissue and are not the natural target cells for ASFV, which primarily infects porcine macrophages. As a result, many viral functions may not be fully recapitulated in this heterologous system, limiting the ability of pE120R to support viral replication. These data suggest that the contribution of pE120R to ASFV replication remains to be fully understood and warrants further exploration in more suitable experimental systems.

In this study, we successfully established a stable HEK293T cell line to express pE120R, providing a controllable and reproducible *in vitro* system. Using this model, we demonstrated for the first time that anti-pE120R antibodies can induce ADCC activities *in vitro*, with a cytotoxicity level of approximately 29%. However, this study has certain limitations. While the observed ADCC activity suggests potential effector functions of the antibodies, the extent to which such *in vitro* responses contribute to effective immune protection *in vivo* remains to be determined. In the future, it will be essential to develop subunit or mRNA vaccines targeting pE120R and to conduct efficacy evaluations.

## Figures and Tables

**Table 1:** Information on different ASFV strains.

Strains	Accession Number	Hosts	Countries	Genotypes
Timor-Leste/2019/1	MW396979.1	Domestic pig	Timor-Leste	II
Georgia/2007/1	FR682468.2	Domestic pig	Georgia	II
Russia/Primorsky 2023	PP348677.1	Wild boar	Russia	II
China/HLJ/2018	MK333180.1	Domestic pig	China	II
China/Henan/2022	OQ504954.1	Domestic pig	China	I
Italy/Sardinia/2018	MW736611.1	Wild boar	Italy	I
China/Inner Mongolia/2022	OQ504955.1	Domestic pig	China	I
China/Jiangsu/2021	OQ504956.1	Domestic pig	China	I/II recombinant
Vietnam/VNUA/2023	PP810980.1	Domestic pig	Vietnam	I/II recombinant

**Table 2:** T-cell and B-cell epitope prediction for pE120R.

Epitopes	Start–End Positions	Peptide Sequences	Scores
CD8^+^ T-cell	142–150	YTDIEMNRL	0.45
CD8^+^ T-cell	48–56	NADTMIPSF	0.45
CD8^+^ T-cell	89–97	AEEEDSNFL	0.37
CD4^+^ T-cell	81–95	TREFTSLVPDEADNK	0.93
CD4^+^ T-cell	80–94	DTREFTSLVPDEADN	0.86
CD4^+^ T-cell	82–96	REFTSLVPDEADNKP	0.78
CD4^+^ T-cell	79–93	HDTREFTSLVPDEAD	0.68
B-cell	9–21	QYLKEDSRDRTSI	None
B-cell	42–57	EEFEPIPDYDPTTSTS	None
B-cell	87–109	LVPDEADNKPEDDEESGAKPKKK	None

**Table 3:** Primers used in this study.

Primers	Sequences (5′-3′)
PLVX-IRES-ZsGreen-F	GAATTCGCCACCATGGCCTTCCTGTGGCTGCTGAGCTGCTGGGCCCTGCTGGGCACCACCTTCGGCGAC
PLVX-IRES-ZsGreen-R	TACAGACATAGAGATGAACCGACTTGGAAAGGGCGGTGGCGGTAGCGGCGGTGGCGGTAGCCACCACCACCACCACCACTAAGAATTC
E120R-V-F	ACCATGGCTTTCCTTTGGCTT
E120R-V-R	ATGTCGGTGTACTTGCTCTTG

## Data Availability

The datasets used and/or analyzed during the current study are available from the corresponding author upon reasonable request.

## Abbreviations

The following abbreviations are used in this manuscript:

ASF	African swine fever
ASFV	African swine fever virus
LAVs	Live-attenuated vaccines
CTLs	Cytotoxic T lymphocytes
PAMs	Porcine alveolar macrophages
PBMCs	Peripheral blood mononuclear cells
LDH	Lactate dehydrogenase
hpi	Hours post-infection
SDS-PAGE	Sodium dodecyl sulfate–polyacrylamide gel electrophoresis
FACS	Fluorescence-activated cell sorting
ADCC	Antibody-dependent cellular cytotoxicity
CDC	Complement-dependent cytotoxicity
ADCP	Antibody-dependent cellular phagocytosis
